# Return of pandemic H1N1 influenza virus

**DOI:** 10.1186/s12879-014-0710-1

**Published:** 2014-12-31

**Authors:** Hilda Sherbany, John McCauley, Tal Meningher, Musa Hindiyeh, Rita Dichtiar, Michal Perry Markovich, Ella Mendelson, Michal Mandelboim

**Affiliations:** Central Virology Laboratory, Ministry of Health, Public Health Services, Chaim Sheba Medical Center, Tel Hashomer, Ramat-Gan, Israel; WHO Collaborating Centre for Reference and Research on Influenza, Division of Virology, MRC National Institute for Medical Research, Mill Hill, London, NW7 1AA UK; The Mina & Everard Goodman Faculty of Life Sciences, Bar-Ilan University, Ramat-Gan, Israel; Israel Center for Disease Control, Ministry of Health, Gertner Institute for Health Policy Research, Chaim Sheba Medical Center, Tel Hashomer, Ramat-Gan, Israel; Department of Epidemiology and Preventive Medicine, School of Public Health, Sackler Faculty of Medicine, Tel-Aviv University, Tel-Aviv, Israel

## Abstract

**Background:**

Influenza pandemics are usually caused by the re-assortment of several influenza viruses, results in the emergence of new influenza virus strains that can infect the entire population. These pandemic strains, as well as seasonal influenza viruses, are subjected to extensive antigenic change that has, so far, prevented the generation of a universal vaccine.

**Methods:**

Samples of patients hospitalized due to infection with the pandemic H1N1 influenza virus (A(H1N1)pdm09) from 2009, when the virus first appeared, until 2013 were analyzed.

**Results:**

While many patients were hospitalized in 2009 due to infection with the pandemic H1N1 influenza virus, only small percentages of patients were hospitalized later in 2010–2012. Surprisingly, however in 2012–2013, we noticed that the percentages of patients hospitalized due to the pandemic H1N1 influenza infection increased significantly. Moreover, the ages of hospitalized patients differed throughout this entire period (2009–2013) and pregnant women were especially vulnerable to the infection.

**Conclusions:**

High percentages of patients (especially pregnant women) were hospitalized in 2013 due to the A(H1N1)pdm09 infection, which may have been enabled by an antigenic drift from those which circulated at the onset of the pandemic.

**Electronic supplementary material:**

The online version of this article (doi:10.1186/s12879-014-0710-1) contains supplementary material, which is available to authorized users.

## Background

Influenza viruses are responsible for most respiratory infections, affecting all age groups, particularly the elderly population [1],[2]. Annual epidemics of seasonal influenza are estimated to result in approximately three to five million cases of severe illness and about 250,000 to 500,000 deaths worldwide (WHO, 2009). The virus contains a single strand RNA molecule which encodes at least 12 proteins; of these proteins, M2, an ion channel protein, neuraminidase (NA) and hemagglutinin (HA) are expressed on the cell surface of infected cells [3].

In influenza virus-infected cells, the assembly and the budding of progeny viruses is the final and critical step in the life cycle of the virus. This step, which significantly affects disease progression [4], requires the coordinated localization of the viral HA and NA proteins to lipid rafts domains on the apical cell membrane [5]-[7]. Membrane localization of HA and NA allows the virus to acquire these glycoproteins by simply budding through the host cell membrane [8]. To complete viral particle budding, the viral NA enzyme cleaves sialic acid residues that link the progeny virus to the infected cells [5].

Because the “foreign” HA and the NA proteins are found on the virus and on the infected cells, they elicit innate, humoral and cellular immune responses, directed against the virus. To avoid this immune attack, both the HA and the NA proteins undergo extensive antigenic changes, complicating design of a universal vaccine that would be effective against all influenza strains. The WHO vaccine that is administered each year is based on the antigenic properties of emerging and circulating strains and contains three influenza virus strains. Nevertheless, every year, millions are infected with influenza.

In addition, influenza pandemics, that occur every few decades, pose a major global threat. Three influenza pandemic outbreaks occurred in the 20th century and one has already been recorded in the current century. The most devastating, was the 1918 Spanish flu pandemic (A/H1N1), which lasted only two years (1918–1919), but took the lives of approximately 50 million people [9]. Two later influenza pandemics, that occurred in 1957 (Asian flu, H2N2) and in 1968 (Hong Kong flu, H3N2), resulted in approximately 70,000 and 34,000 deaths, respectively, in the US alone [1]. The most recent influenza (A(H1N1)pdm09) pandemic emerged in 2009. Although the mortality rate was relatively low, many people around the world were infected by the virus [9]. The emergence of new pandemic in recent years provides us with a unique opportunity to study the virus spread and evolution through out the years from the time it first appeared until today.

Here, we analyzed data obtained from patients hospitalized in the period between May 2009 and May 2013 due to A(H1N1)pdm09 infection. We observed that in 2013 a significant number of patients (including increasing percentages of pregnant women) were hospitalized due the A(H1N1)pdm09 infection and we suggest that the virus re-appeared because of antigenic changes it acquired over the last 4 years.

## Methods

### Ethics

This is a retrospective study performed on anonymous patient samples that were analyzed for the presence of various viruses, as part of the routine tests performed in the Sheba Medical Center. No extra samples were obtained for this research. Therefore, informed consent was not required. The institutional review board (IRB) of the Sheba Medical Center approved this research (Helsinki Number 0402-13-SMC).

### Patients and samples

Respiratory clinical samples (nasopharyngeal swabs or aspirates) were collected from 29,956 patients hospitalized at Chaim Sheba Medical Center, Israel, due to respiratory illnesses, during the winter seasons between May 2009 and May 2013. 4,674 patients were positive for A(H1N1)pdm09 virus infection. All patients were from the same geographical area.

### Detection of A(H1N1)pdm09 virus infection

Viral genomic RNA was extracted from patient samples by using the NucliSENS easyMAG (BioMerieux, France). A(H1N1)pdm09 infection was detected by using a panel of real-time reverse transcription-PCR (rRT-PCR), as previously described [10],[11].

### Phylogenetic analysis

For the phylogenetic analysis, influenza hemagglutinin protein-specific primers were used by WHO Swine genome set [12] (Table 1). The Sequencher® 5.0 program (Gencodes Corporation, Ann Arbor, MI) was used to compare the nucleotide sequences. Phylogenetic trees were prepared by nearest neighbor joining analysis using Clustal X with 1000 bootstraps and trees were visualized using TreeView or NJ plot software. The sequences were submitted to the NCBI.

**Table 1:** **Primer sequences**

Fragment	HA forward	Primers 5'-3'	HA reverse	Primers 5'-3'
**Fragment 1**	1	TGT AAA ACG ACG AGT ATA CGA CTA GCA AAA GCA GGG G	461	CAG GAA ACA GCT ATG ACC TCA TGA TTG GGC CAY GA
**Fragment 2**	351	TGT AAA ACG ACG GCC AGT ACR TGT TAC CCW GGR GAT TTC A	943	CAG GAA ACA GCT ATG ACC GAA AKG GGA GRC TGG TGT TTA
**Fragment 3**	379	TGT AAA ACG ACG GCC AGT ACR TGT TAC CCA GGR GAT TTC	1204	CAG GAA ACA GCT ATG ACC TCT TTA CCY RCT GTG AA
**Fragment 4**	736	TGT AAA ACG ACG GCC AGT AGR ATG RAC TAT TAC TGG AC	1340	CAG GAA ACA GCT ATG ACC TTC TKC ATT RTA WGT CCA AA
**Fragment 5**	1124	TGT AAA ACG ACG GCC AGT TGG ATG GTA YGG TTA YCA YCA	1541	CAG GAA ACA GCT ATG ACC TCA TAA GTY CCA TTT YTGA
**Fragment 6**	1204	TGT AAA ACG ACG GCC AGT AAG ATG AAY ACR CAR TTC ACAG	1778	CAG GAA ACA GCT TCA GTA GAA ACA ACA AGG GTG TTT

### Statistical analysis

The positivity percent was calculated by dividing the number of positive H1N1pdm samples by the total number of tests performed in the laboratory. A Binomial 95% confidence interval was calculated for this rate. The chi-square test was applied to evaluate the differences in positivity percent between the compared years and between the different age groups within the same year. A p value <0.05 was considered to be statistically significant.

**Figure 1:**
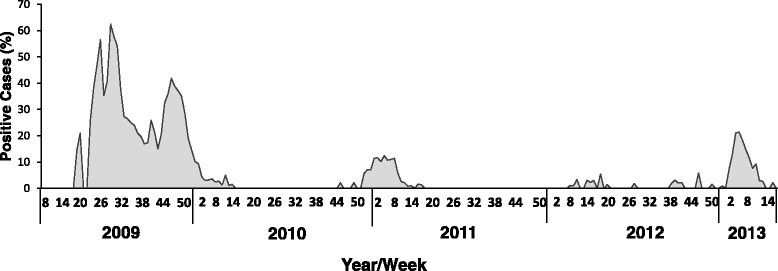
**Distribution of H1N1 pandemic virus infection in hospitalized patients.** The percentage of patients hospitalized (on a weekly basis, X axis) due to influenza-like syndrome and infected with the pandemic 2009 influenza virus.

**Figure 2:**
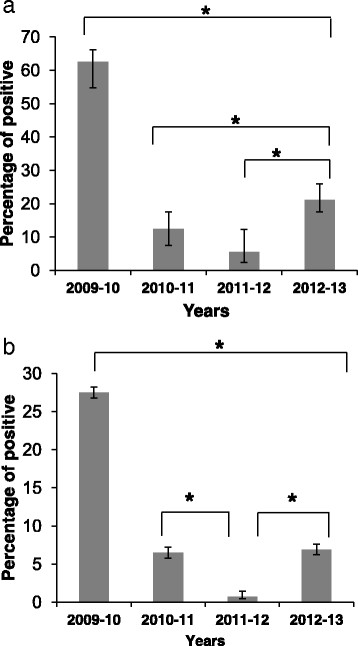
**Infection peaks and annual infection percentages.** The figure shows the percentages of patients infected with the pandemic 2009 influenza virus between 2009 and 2013. The percentages of patients hospitalized due to influenza-like syndrome are presented either at the peak of the infection in each year **(a)** or as an annual average of the infection **(b)**. *P < 0 · 05 using the chi-square test.

**Figure 3:**
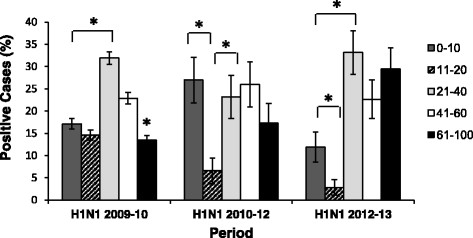
**Age distribution of the infected patients.** The age percentages of patients hospitalized due to influenza-like syndrome and infected with the pandemic 2009 influenza virus. Three periods of infection are presented (X axis). *P < 0 · 05 using the chi-square test.

All analyses were performed using SPSS (version 21.0.0. SPSS Inc., Chicago, IL, USA), SAS (SAS 9.1, SAS Institute Inc, Cary, NC, USA) and Excel softwares.

## Results

### Rebound of pandemic H1N1 influenza

We demonstrate in this paper that the pandemic H1N1 influenza virus reappeared in Israel in 2012–2013. We noticed that the percentages of patients hospitalized due to the pandemic H1N1 influenza infection increased significantly in this year. Interestingly, we also observed that pregnant women were especially vulnerable to the infection. We have started this research by analyzing the percentages of patients infected with the A(H1N1)pdm09 virus from the time it first appeared in the country until 2013. The new influenza A(H1N1)pdm09 virus was observed in Israel throughout the entire year of 2009 following its initial identification in May 2009. A(H1N1)pdm09-infected patients were hospitalized from May 2009 until the virus ceased to be prevalent, at the end of the first quarter of 2010 (Figure 1). From the end of 2010 until the beginning of 2011, relatively fewer patients were hospitalized due to the A(H1N1)pdm09 infection. Only sporadic cases of A(H1N1)pdm09 infection were reported in the winter season of 2011–12. However, surprisingly, at the beginning of 2013, the percentages of patients hospitalized due to A(H1N1)pdm09 infection increased dramatically (Figure 1).

**Figure 4:**
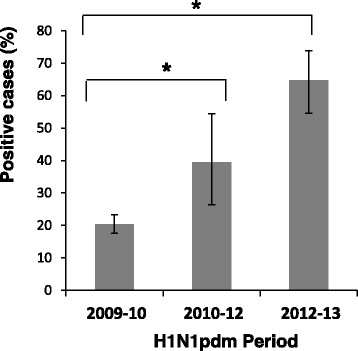
**The percentage of positive cases for pregnant women.** The percentages (out of the 21–40 age group) of pregnant women hospitalized due to influenza-like syndrome and infected with the pandemic 2009 influenza virus from 2009-today (2013). Three periods of infection are presented (x axis). *P < 0 · 05 using the chi-square test.

Figure 2 summarizes the infection peaks and the annual percentages of patients infected with A(H1N1)pdm09 virus. As can be seen, in 2009–10, when the virus first appeared, approximately 60% of the patients admitted with respiratory disease at week 29 of the year were infected with the virus strain. In the following years, infection rates dropped significantly and in 2011–12 (week 16), at the peak of infection for that year, only 5% of the patients were infected with A(H1N1)pdm09 (Figure 2a) and less than 1% of patients were hospitalized due to A(H1N1)pdm09 infection throughout the entire year (Figure 2b). In week 6 of 2012–13, a dramatic increase in the percentages of hospitalized patients was observed, with around 20% of the patients presenting infection with the A(H1N1)pdm09 virus (Figure 2a). Similarly, the annual percentages of patients hospitalized due to the A(H1N1)pdm09 infection was higher as compared to 2011–2012 (Figure 2b).

To investigate the reasons accounting for the return of the pandemic H1N1 influenza virus in 2013, we analyzed the ages of the infected patients over three periods: when the virus first appeared (2009–10), when only a low percentage of patients were infected with the virus (2010–12; the age of the patients in each year was not analyzed separately due to the small number of patients hospitalized in these years) and at 2013, when a relatively high percentage of patients was hospitalized due to the A(H1N1)pdm09 infection. In 2009–10, most of the infected patients were 21–60 years of age (Figure 3 and Table 2). In 2010–12, equal infection rates were observed throughout all age groups, with the exception of adolescent patients, aged 11–20 (Figure 3 and Table 2). Similarly, in 2012–13, patients 11–20 years of age were less affected by the A(H1N1)pdm09 virus when compared to other age groups. However, in 2012–13, most of the A(H1N1)pdm09-infected patients were 21–40 or 61–100 years of age group.

**Table 2:** **Male and female patient distribution**

Age		H1N1pdm 2009–10 (1y)	H1N1pdm 2009–12 (1y)	H1N1pdm 2009–13 (1y)	P value
0-10	Patients n	5112	3001	1367	
	Positive n (%)	879 (17.2)	80.(2.7)	51 (3.7)	<.0001
	Male n (%)	2908 (56.9)	1784 (59.4)	780 (57.1)	0.0682
	Male Positive n (%)	499 (17.2)	39 (.2)	24 (3.1)	<.0001
	Female n (%)	2204 (43.1)	1217 (40.6)	587 (42.9)	0.0682
	Female positive n (%)	380 (17.2)	41 (3.4)	27 (4.6)	<.0001
11-20	Patients n	1621	390	205	
	Positive n (%)	733 (45.2)	20 (5.1)	10 (4.9)	<.0001
	Male n (%)	927 (57.2)	197 (50.5)	113 (55.1)	0.0573
	Male Positive n (%)	412 (44.4)	11 (5.6)	6 (5.3)	<.0001
	Female n (%)	694 (42.8)	193 (49.5)	92 (44.9)	0.0573
	Female positive n (%)	321 (46.3)	9 (4.7)	4 (4.3)	<.0001
21-40	Patients n	3888	990	800	
	Positive n (%)	1606 (41.3)	74 (7.5)	121 (15.1)	<.0001
	Male n (%)	1978 (50.9)	481 (48.6)	401 (50.1)	0.4317
	Male Positive n (%)	828 (41.8)	31 (6.44)	30 (7.48)	<.0001
	Female n (%)	1910 (49.1)	509 (51.4)	399 (49.9)	0.4317
	Female positive n (%)	778 (40.7)	43 (8.44)	91 (22.8)	<.0001
	**Pregnant positive n (%)**	**158 (20.3)**	**17 (39.5)**	**59 (64.8)**	<.0001
41-60	Patients n	3033	928	864	
	Positive n (%)	904 (29.8)	76 (8.2)	80 (9.3)	<.0001
	Male n (%)	1563 (51.5)	503 (54.2)	507 (58.7)	0.008
	Male Positive n (%)	464 (29.7)	43 (8.5)	38 (7.5)	<.0001
	Female n (%)	1470 (48.5)	425 (45.8)	357 (41.3)	0.008
	Female positive n (%)	440 (29.9)	33 (7.8)	42 (11.8)	<.0001
61-100	Patients n	4497	1975	1872	
	Positive n (%)	378 (8.4)	50 (2.5)	105 (5.6)	<.0001
	Male n (%)	2550 (56.7)	1063 (53.8)	976 (52.1)	0.0015
	Male Positive n (%)	189 (7.4)	28 (2.6)	58 (5.9)	<.0001
	Female n (%)	1942 (43.2)	912 (46.2)	896 (47.9)	0.0015
	Female positive n (%)	189 (9.7)	22 (2.4)	47 (5.2)	<.0001

We included in this table only patients that we were able to define by gender. In bold are the pregnant women.

### Pregnant women are particularly sensitive to A(H1N1)pdm09 infection

We next investigated the percentages of males and females that were hospitalized due to A(H1N1)pdm09 virus infection. Particular focus was placed upon the 21–40 age group, as in 2013, this group was particularly affected by the A(H1N1)pdm09 virus. The highest number of patients (between the ages of 21–40) hospitalized due to influenza symptoms was observed in 2009–10, when the A(H1N1)pdm09 first appeared (Table 2), with similar prevalence among males and females (Table 2). In 2010–12, fewer patients were hospitalized with influenza-like illness, which equally affected both males and females (Table 2). In contrast, in 2013, when a new wave of A(H1N1)pdm09 virus infection arrived, the number and the percentages of women hospitalized due to A(H1N1)pdm09 infection were much higher than those of the men (Table 2). More specifically, the percentages of pregnant women hospitalized due to the pandemic H1N1 influenza virus infection gradually increased from 2009 until 2013, accounting for <60% of the women (ages 21–40) infected in 2013 (Figure 4). A simple logistic regression analysis (Table 3) showed that within the group of pregnant women, no differences in the ages of the infected women were noticed throughout the year, however, as we demonstrate here, differences in the increased percentages of infected women were observed in 2012–13.

**Table 3:** **Logistic regression**

Variable	Odds ratio	p-value
Period 2009–10 (Reference)	1	
Period 2010-12	(1.33-4.77) 2.52	0.0045
Period 2010-13	(4.53-11.5) 7.21	<.0001
Age (as continuous)	1.01 (0.99-1.04)	0.3486

### Circulation of genetic variants of the pandemic H1N1 influenza virus strains

**Figure 5:**
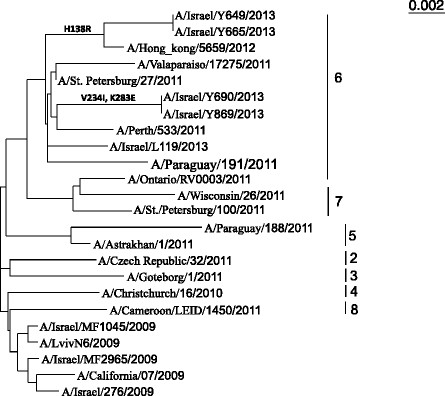
**Phylogenetic tree.** Phylogenetic tree was generated using the primers listed in Table 1. The HA genes of H1N1 viruses cluster into eight genetic groups defined by amino acid substitution in HA1. The numbers indicate the different subgroups of the pandemic influenza viruses. Mutations are indicated.

The differences in the ages of the hospitalized patients observed throughout the years and the rebound of the A(H1N1)pdm09 virus infection observed in 2012–13, led us to hypothesize that the A(H1N1)pdm09 virus had undergone changes during this short period. Several influenza viruses were isolated from the samples collected during these years, to amplify their hemagglutinin gene using the primers described in Table 1. The obtained sequences were used to build a phylogenetic tree. Viruses isolated in 2009 and in 2013 diverged differently in the phylogenetic tree (Figure 5). While the influenza strains obtained in 2009 resemble the strain used for the influenza vaccine of that year, those who were isolated in 2011–13 resembled other strains, in particular, group 6 viruses.

## Discussion and conclusions

Influenza pandemics that occur every few decades put the entire population at risk [13], and present a huge economic burden. The last influenza pandemic, which occurred in 2009 and was caused by the A(H1N1)pdm09 virus, is the second documented pandemic involving H1N1 influenza viruses. The first one was the 1918 influenza pandemic which killed tens of millions of people [9]. The 2009 influenza virus resulted from triple reassortment of bird, swine and human influenza viruses further combined with a Eurasian pig flu virus [14].

As shown here and by others [15], at the beginning of 2009, unlike most strains of influenza viruses [16], adults older than 60 years of age were less affected by the A(H1N1)pdm09 virus [17], while the most affected group of patients were within the 21–40 year-old age. In contrast, we show here, as far as we know, for the first time, that when the virus returned in 2013, that in addition to the 21–40 year-old adults, adults over 60 years of age were particularly sensitive to infection. The reasons accounting for the age-dependent sensitivity of the different age groups are unknown. Moreover, more women were hospitalized in 2013 due to A(H1N1)pdm09 virus infection, which we attribute to the increased hospitalization of pregnant women observed in 2013. Already in 2009, when the virus first appeared, pregnant women and obese individuals were shown to be more susceptible to infection [18],[19]. In 2009–2010 when the A(H1N1)pdm09virus first appeared other influenza virus strains were hardly detected [20],[21]. In later years infections with the following H3N2 viruses A/Perth/16/2009-like and A/Victoria/361/2011-like were detected. Furthermore, the pattern of respiratory virus infections (other than influenza) changed during this time period, especially around 2009 [22]-[24]. We don’t think however that this is the reason of why more pregnant women were hospitalized in 2013 due to the A(H1N1)pdm09 infection. Thus, it is currently unknown why pregnant women are more sensitive to infection with the pandemic virus.

The 2009 pandemic influenza began in Mexico [9]. The Mexican government closed most of Mexico City's public and private facilities in an attempt to contain the spread of the virus; however, it continued to spread globally. In June 2009, the World Health Organization (WHO) and the U.S. CDC stopped counting cases of people infected with the virus and WHO declared the outbreak of a pandemic.

As reported here and by others [25],[26], the pandemic declined around the world at the beginning of 2010. In August 2010, the Director-General of the WHO announced the end of the H1N1 pandemic [27], further informing that the pandemic influenza virus had moved into the post-pandemic period and could be considered a seasonal influenza virus. Today, experts, including at the WHO, have agreed that an estimated 284,500 people were killed by the disease, much higher than the initial estimates of the death toll. We demonstrated here, however, that the virus did not disappear, as reported by others as well [28].

However, we submit that the risk of infection with the H1N1pdm09 virus should still be taken seriously and that its ability to cause severe disease has not abated: while in 2010–2012 the virus was detected at low percentages in hospitalized patients, in 2013, infections with the pandemic influenza virus increased dramatically and affected a variety of age groups. We suggest that genetic alterations, which resulted in reduced viral recognition by antibodies generated against the 2009 pandemic influenza virus, underlie the return of the A(H1N1)pdm09 virus in 2013. This is despite observations, we made previously in which we show that around half of the Israeli population had antibodies against A(H1N1)pdm09 [29]. Nevertheless, the fact that the virus, in 2013, primarily infected elderly individuals and people at the ages between 21–40, support the claim that it transformed from a pandemic virus to a seasonal influenza virus. Still, the dramatic increase in the percentages of hospitalized patients, especially pregnant women, in 2013, suggests that the threat from the pandemic virus still exists.

## Authors’ original submitted files for images

Below are the links to the authors’ original submitted files for images. Authors’ original file for figure 1 Authors’ original file for figure 2 Authors’ original file for figure 3 Authors’ original file for figure 4 Authors’ original file for figure 5
