# Supplementary figures and images for: Return of pandemic H1N1 influenza virus
Source: BMC Infect Dis. 2014 Dec 31;14:710. doi: 10.1186/s12879-014-0710-1 (PMC4375933; doi:10.1186/s12879-014-0710-1)

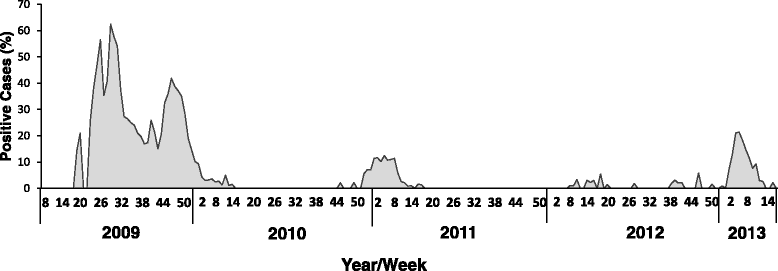

Supplement: Supplementary file 1 — Authors’ original file for figure 1 [file 12879_2014_710_MOESM1_ESM.gif]

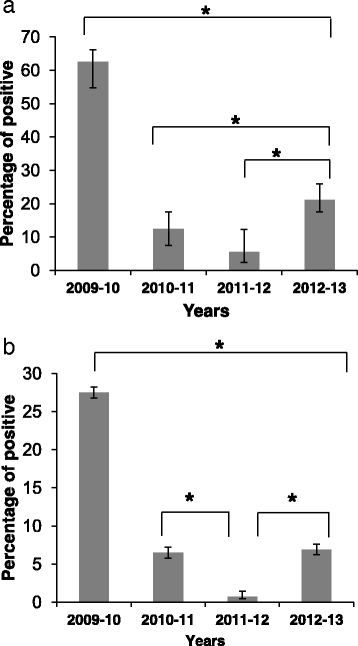

Supplement: Supplementary file 2 — Authors’ original file for figure 2 [file 12879_2014_710_MOESM2_ESM.gif]

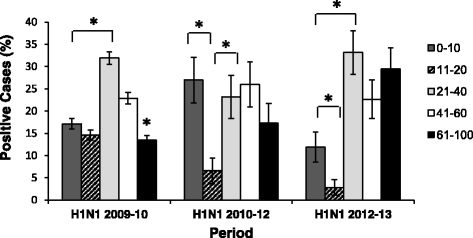

Supplement: Supplementary file 3 — Authors’ original file for figure 3 [file 12879_2014_710_MOESM3_ESM.gif]

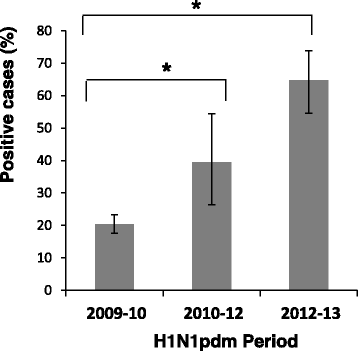

Supplement: Supplementary file 4 — Authors’ original file for figure 4 [file 12879_2014_710_MOESM4_ESM.gif]

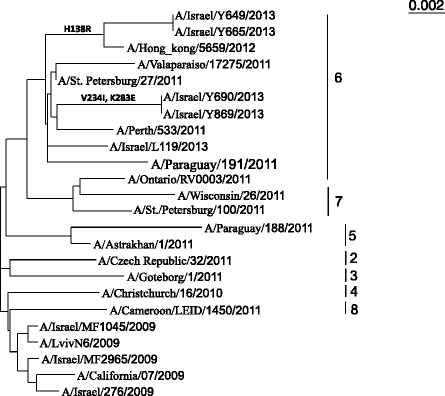

Supplement: Supplementary file 5 — Authors’ original file for figure 5 [file 12879_2014_710_MOESM5_ESM.gif]
